# First Record of *Amphitrite cirrata* (Polychaeta: Terebellidae) in Association with the Barents Sea Red King Crab *Paralithodes camtschaticus* (Malacostraca: Lithodidae)

**DOI:** 10.3390/ani16010078

**Published:** 2025-12-26

**Authors:** Alexander G. Dvoretsky, Vladimir G. Dvoretsky

**Affiliations:** Murmansk Marine Biological Institute of the Russian Academy of Sciences (MMBI RAS), 183038 Murmansk, Russia

**Keywords:** *Amphitrite cirrata*, red king crab, association, Barents Sea

## Abstract

The introduced red king crab *Paralithodes camtschaticus* holds significant commercial value in the Barents Sea. This species is recognized as a host for a wide variety of symbiotic organisms, including polychaetes. In July 2015 and 2025, a total of 12 specimens of the marine terebellid polychaete *Amphitrite cirrata* were discovered inhabiting the gills of two red king crabs in Dalnezelenetskaya Bay, Barents Sea. This study represents the first documented occurrence of an association between these benthic species. Colonization of the red king crab by *Amphitrite cirrata* offers several advantages to the polychaetes by providing access to suitable feeding conditions, increased mobility, and protection from potential predators. However, this association poses disadvantages to the host crabs, as it results in tissue damage and an elevated concentration of sand particles within their gills.

## Simple Summary

This study reports the first documented association of the marine polychaete *Amphitrite cirrata* with the red king crab (*Paralithodes camtschaticus*) in the Barents Sea. Previously known as a widely distributed tube-building worm inhabiting various marine substrates, *Amphitrite cirrata* has not been recorded as an associate of marine animals. This finding sheds new light on the evolving symbiotic relationships in the red king crab’s introduced range. The worms were found in the gills of red king crabs, causing tissue damage and contamination with sand and organic waste, which may impair the crab’s respiration and molting abilities. While *Amphitrite cirrata* benefits from the host environment through access to food, protection, and dispersal, it represents a potential parasitic threat to host health.

## 1. Introduction

The Barents Sea is a highly productive region due to the interaction between cold Arctic and warm Atlantic waters, as well as the presence of a wide range of environmental conditions [1,2]. Although this region is characterized by high benthic diversity [3], it lacks any abundant, commercially important native crab species [4]. The red king crab, *Paralithodes camtschaticus* (Tilesius, 1815), is a large commercially valuable species that was intentionally introduced into the Barents Sea from the North Pacific Ocean between 1961 and 1969 to establish a new local fishery [5]. The population of red king crabs in the Barents Sea exhibited a dramatic increase from 1995 to 2003, rising from an estimated 2500 individuals to over 600,000 [6]. However, a subsequent decline in landings was recorded between 2005 and 2011, decreasing from 13,100 metric tons to 3702 metric tons. Following 2010, positive trends in the red king crab commercial stock have been observed, with landings increasing from 4000 to 5000 metric tons in 2010–2012 to 5500–6400 metric tons in 2013–2015, eventually reaching 8300 metric tons in 2016 [7]. The most recent data indicate an annual landing of 10,420 metric tons [8,9]. These dynamics have been shown to be driven by climatic factors, fishing pressure, and changes in the management of the red king crab in the region [9,10,11,12].

The population dynamics, distribution, reproduction, feeding, molting, limb injury, behavior [13,14,15,16,17,18,19,20], and associations with symbiotic organisms of *Paralithodes camtschaticus* have been the focus of extensive research over the past several decades [21,22,23,24]. Within its novel habitat in the Barents Sea, the red king crab has become a host for various local benthic species, including parasites, commensals, and other epibionts [25,26]. Annual surveys conducted by the Murmansk Marine Biological Institute (MMBI), utilizing consistent sampling methods and processing protocols, have substantially contributed to our understanding of the organisms that interact with this invasive crab species in its introduced range [8,27,28].

In this study, we report, for the first time, the association of the marine terebellid polychaete *Amphitrite cirrata* Müller, 1776 with the red king crab in the Barents Sea.

## 2. Materials and Methods

**Figure 1:**
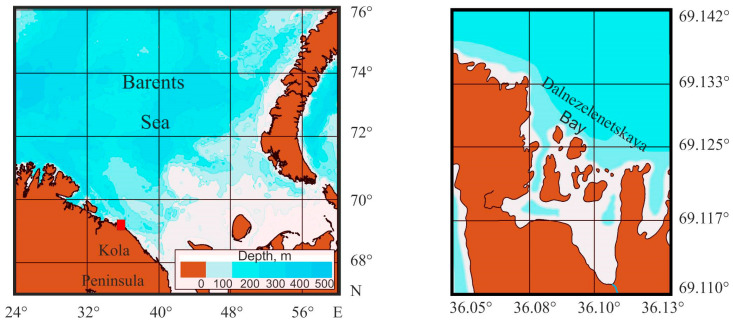
Map of the study area, with a red square indicating the location of Dalnezelenetskaya Bay in the coastal Barents Sea.

Red king crabs were collected by divers from depths of 15–30 m in Dalnezelenetskaya Bay (Figure 1), a small semi-enclosed gulf in Eastern Murman [29,30], in July 2015 and 2025.

For each crab, sex, weight, and shell condition were assessed following the methodology described by Donaldson and Byersdorfer [31] and Dvoretsky and Dvoretsky [17]. Old-shell crabs display partial or total brown scratching on the ventral coxa and have muscle-filled, firm legs. Very old-shell crabs exhibit dense, dark scratching and rounded dark dactyls; their legs remain full of muscle and are difficult to compress. Carapace length (CL) was measured to the nearest millimeter from the posterior margin of the right eye socket to the midpoint of the rear margin of the carapace [19].

In the laboratory of the MMBI biological station in Dalnezelenetskaya Bay, the body of each crab—including the carapace, appendages, abdomen, mouthparts, and branchial chambers—was examined for associated species. The gills were removed and preserved in 4% formalin and subsequently analyzed under a stereomicroscope. Since the samples were processed immediately after the expedition was completed, formalin fixation did not affect the detectability or morphology of symbionts. All individual symbiotic organisms encountered were identified and enumerated [32]. The methodologies followed adhere to those described in previous studies [27,28].

## 3. Results

A total of 53 and 11 red king crabs were collected from Dalnezelenetskaya Bay to investigate symbionts and epibionts living on or within the crabs in 2015 and 2025, respectively. Among these crabs, eight species of polychaetes were detected. For the first time, the marine terebellid polychaete *Amphitrite cirrata* Müller, 1776 was documented as an associate of *Paralithodes camtschaticus*. Three crabs out of the sample set were found to harbor *Amphitrite cirrata*, corresponding to a symbiotic infestation rate of 4.7% (3.8% in 2015 and 9.1% in 2025). Key morphometric and other characteristics of these host crabs are summarized in Table 1.

**Figure 2:**
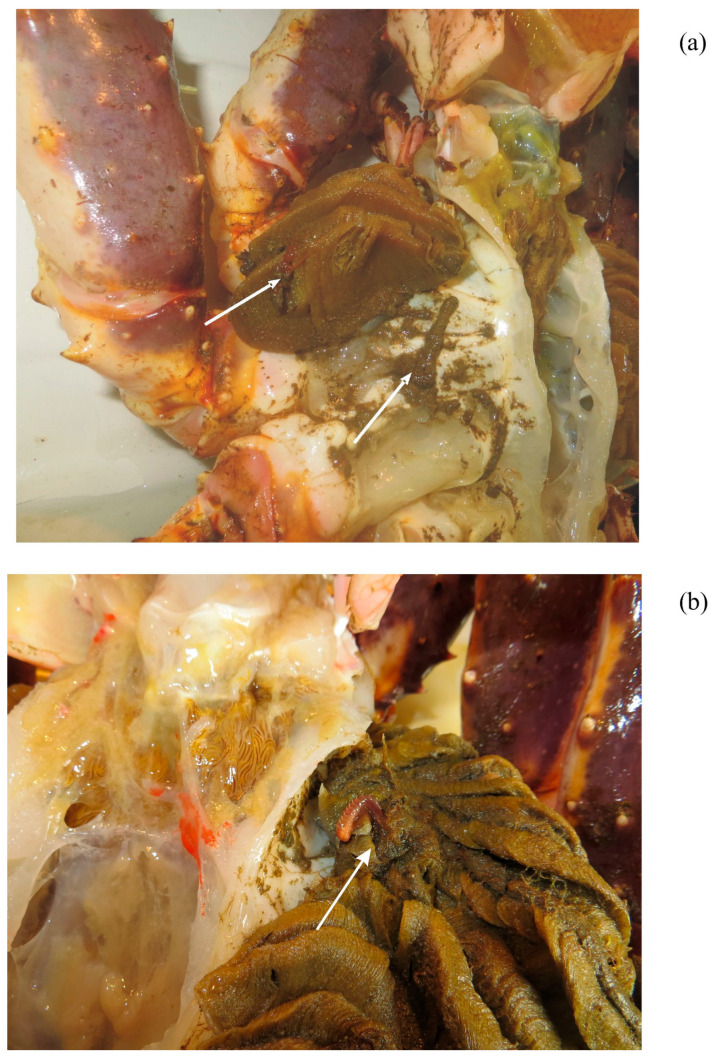
Photograph of *Amphitrite cirrata* infestations on *Paralithodes camtschaticus* gills from Dalnezelenetskay Bay. (**a**)—2015 (crab 1), (**b**)—2025 (Crab 3).

In both cases where *Amphitrite cirrata* was detected, the worms were located in the gills of the host crabs (Figure 2).

The gills infested by *Amphitrite cirrata* contained substantial concentrations of sand particles and exhibited visible damage to crab tissues including erosions and lesions. In contrast, the gills of non-infested old-shell crabs showed no such damage.

Accompanying the worms, other symbiotic organisms were identified. The most common associates included copepods such as *Harpacticus chelifer* (O.F. Müller, 1776), *Harpacticus flexus* Brady and Robertson D., 1873, and *Tisbe furcata* (Baird, 1837), as well as the amphipod *Ischyrocerus commensalis* Chevreux, 1900. The intensity of infestation of these associated species on crabs colonized by *Amphitrite cirrata* ranged from 84 to 1920 individuals per crab.

## 4. Discussion

The discovery of *Amphitrite cirrata* associated with red king crabs in the Barents Sea represents the first documented case of a relationship between this polychaete species and an invertebrate host. *Amphitrite cirrata* is a tube-building worm widely distributed in various marine environments, including sandy and muddy substrates, on rhizoids of *Laminaria* or *Saccharina*, and on hard-bottom substrates such as coral reefs and Sabellariidae reefs [33,34]. It inhabits depths ranging from the intertidal zone to over 2700 m [35]. Its geographic range includes the Mediterranean, Baltic, Norwegian, Barents, White, Kara, and Chukchi Seas, as well as the North Pacific [33,35,36,37,38,39]. However, no prior evidence of *Amphitrite cirrata* forming associations with other marine crustaceans [40,41,42,43], including red king crabs in their native range [44], has been reported.

Previous surveys [8,23] documented 12 polychaete species living on red king crabs in Dalnezelenetskaya Bay. Among these, the highest infestation rates were observed for *Harmothoe imbricata* (L., 1767) (17.7% and 13.3% incidence at shallow and deep sites, respectively) and *Circeis armoricana* (Saint-Joseph, 1894) (7.2% and 17.8%, respectively). The relatively low prevalence of *Amphitrite cirrata* (3.8%) in this study suggests a facultative relationship rather than a strict obligate one [45].

The discovery and ongoing findings of new symbiotic species associated with red king crabs in their novel habitat [8,24,27] indicate that the symbiotic community surrounding *Paralithodes camtschaticus* is still evolving in the Barents Sea. The dynamics of this assembly process are likely influenced by factors such as fisheries activities and climatic variations, both of which affect red king crab biology and distribution [10,11].

The presence of *Amphitrite cirrata* in the host gills implies that infestation likely occurs through the settlement of planktonic larvae [46]. Observations of a diverse size range of polychaetes on the host crabs point to multiple generations of worms inhabiting the same host individuals [47]. This is consistent with the fact that both crabs hosting *Amphitrite cirrata* in this study had old shells, indicative of an age greater than 12 months [31].

As with many other terebellids, *Amphitrite cirrata* is a tentaculate deposit feeder [37]. Its diet primarily consists of detritus and microalgae, although predation on smaller polychaetes has been documented [35,37]. The combination of water circulation within the host gills, abundant food sources such as detritus and small copepods/amphipods, and protection from predators makes the branchial chambers of *Paralithodes camtschaticus* a favorable microhabitat for *Amphitrite cirrata*. However, the presence of these worms may harm their host, as evidenced by gill tissue damage, sand accumulation, and waste deposits (e.g., fecal pellets). Such factors could impair respiration and reduce the likelihood of successful molting, similar to impacts observed with other symbionts on *Paralithodes camtschaticus* [22].

## 5. Conclusions

Our study marks the first record of *Amphitrite cirrata* associating with an invertebrate host, specifically *Paralithodes camtschaticus*. With this addition, the number of polychaete species colonizing red king crabs in the Barents Sea now totals 13. Given the negative effects of *Amphitrite cirrata* on host gill tissues observed in this study, high infestation intensities may warrant classifying *Amphitrite cirrata* as a parasitic species in this context. Further research is necessary to understand the ecological implications and dynamics of this novel association.

## Figures and Tables

**Table 1:** Characteristics of the three *Paralithodes camtschaticus* specimens colonized by *Amphitrite cirrata* in Dalnezelenetskay Bay.

Parameter	Crab 1	Crab 2	Crab 3
Collection date	6 July 2015	9 July 2015	2 July 2025
Depth, m	18	30	19
Coordinates	69°07′03″ N, 36°04′23″ E	69°07′33″ N, 36°05′26″ E	69°07′02″ N, 36°04′26″ E
Crab sex	Male	Female	Male
Carapace length, mm	165.6	156.0	152.6
Weight, g	3822	3126	3261
Shell condition	Very old shell	Old shell	Old shell
Number of *Amphitrite cirrata*	9	2	1
Size range of worms, mm	28–53	45–56	52

## Data Availability

The original contributions presented in this study are included in the article. Further inquiries can be directed to the corresponding authors.
